# Do Prisoners With Reintegration Needs Receive Relevant Professional Assistance?

**DOI:** 10.1177/0306624X221086554

**Published:** 2022-04-18

**Authors:** Amanda J. Pasma, Esther F. J. C. van Ginneken, Hanneke Palmen, Paul Nieuwbeerta

**Affiliations:** 1 Leiden University, The Netherlands

**Keywords:** prison, reentry, needs, reintegration, professional assistance, support

## Abstract

Ex-prisoners often experience barriers to successful transition regarding employment, finances, housing, healthcare, and valid identification. Based on the Offender Management framework, assistance during imprisonment by prison- and community-based professionals is considered key in preparing prisoners for release regarding these reintegration needs. Therefore, the current study examines the degree to which prisoners with reintegration needs are assisted by relevant professionals. We used self-reported data from 4,309 prisoners of the Dutch Prison Visitation Study, part of the Life in Custody Study. The results showed that prisoners have more contact with prison-based than with community-based professionals, but that the latter relatively often have contact with prisoners with related reintegration needs. Yet, a specific group of prisoners with reintegration needs remains invisible. Prisoners with complex, health, or valid identification needs, and prisoners in the start or pre-release phase require further attention. It is discussed what can be learned from these findings on Dutch Offender Management practices.

Transitioning back to society can be a challenging event for prisoners (Petersilia, 2003; Visher & Courtney, 2007). Upon release, more than 70% of Dutch prisoners are unemployed and more than half encounter financial difficulties (Beerthuizen et al., 2015; Ramakers, 2014). Moreover, one third of Dutch prisoners find themselves in unstable housing situations or experience drug related problems (Den Bak et al., 2018; Wensveen et al., 2016) and 15% do not possess valid identification (Weijters et al., 2018). Similar problems are reported worldwide (e.g., Abbott et al., 2016; McSweeney & Hough, 2006; Visher et al., 2004). These unmet *reintegration needs* form barriers to successful transition (Graffam & Hardcastle, 2007; Visher & Courtney, 2007) and enhance the likelihood of recidivism (Visher et al., 2004, 2017).

According to the Offender Management (OM) framework, support by and cooperation between prison-based and community-based professionals is vital in overcoming these transitioning problems. In most prison institutions, prison-based professionals such as case managers or mentors are primarily in charge of preparing prisoners for release. These prison-based professionals often take care of intake assessments, keep track of the reintegration needs and refer prisoners to specialized help from community-based professionals (Day et al., 2012; Hardyman et al., 2004). In turn, these community-based professionals, among whom parole officers, municipal officers, health- and care professionals, and volunteers, can provide further access to community resources and help prisoners prepare for release. For example, parole officers and municipal officers can assist in employment, finances, housing, or valid identification (Bares & Mowen, 2020; Viglione et al., 2015), healthcare professionals can take care of discharge planning and continuation of healthcare upon release (Hopkin et al., 2018) and volunteers can help with social services, such as housing, debt counseling, and job training (McSweeney & Hough, 2006; O’Connor & Bogue, 2010).

In theory, then, professional support can help prepare prisoners for release, but in practice this appears challenging. Prisoners often report a lack of professional assistance (Crewe & Ievins, 2021; Hamilton & Belenko, 2016; Loeliger et al., 2018), no intake or needs assessments (Hamilton & Belenko, 2016), no in-prison access to community resources (Lloyd et al., 2015; McCauley & Samples, 2017), poor pre-release programing (McCauley & Samples, 2017), or a lack of collaboration between prison-based and community-based professionals in throughcare and aftercare (Abbott et al., 2016; Lloyd et al., 2015; Smith et al., 2018). Although this overall picture of professional assistance in prisons is bleak, these studies do not give information about the extent to which assistance is offered in relation to specific reintegration needs. For example, does a prisoner with employment needs actually receive assistance from a professional who can help with finding employment? It is important to examine the relationship between needs and assistance, because unassisted needs can be problematic for prisoners and for post-release outcomes.

Therefore, the current study aims to examine the degree to which reintegration needs of prisoners are met with support from prison-based and community-based professionals. More specifically, we are interested in (1) how many prisoners with reintegration needs report *any assistance* by prison-based or community-based professionals; (2) the extent to which *specific needs* are related to assistance by *relevant professionals*; and (3) the extent to which the *overall level of needs* is related to the *overall level of assistance*. Finally, because continuity of care is considered crucial in preparing prisoners for release, as we describe later on, we also examine (4) in which *phases of imprisonment* prisoners with needs report assistance.

The following discussion of professional assistance draws on the OM framework. First, we describe the core principles of OM and what good rehabilitation practices in prison should look like accordingly. Second, we describe how these OM principles become visible within the Dutch rehabilitation policy. Based on previous research we then evaluate the degree to which professional assistance usually matches these OM principles. The results of the current study, based on survey data among 4,309 Dutch prisoners, can inform improvement of reintegration support in the Netherlands and beyond.

## Offender Management in Prisons

Across the world, reintegration support in prisons is offered in line with Offender Management (OM) principles (ICPR, 2011; Maguire & Raynor, 2017). Offender management is rooted in *case management* in other human services fields and refers to the general idea that clients have a set of complex needs that should be managed by multiple agencies (ICPR, 2011). In relation to prison sentences, OM strategies aim to manage the needs of offenders, during and after imprisonment, to provide support, and reduce crime. The core principles of OM in prisons can be summarized as follows: (1) a prison-based case manager coordinates the reintegration process; (2) collaboration with/early involvement of community-based agencies is necessary to provide access to resources; (3) continuity of care throughout and after the whole sentence is important; (4) the focus should be on the individual needs of offenders, rather than on general treatments for certain types of offenders; and (5) personal relationships between prisoners and professionals are key (Maguire & Raynor, 2017).

Most OM perspectives share these core principles, but they often slightly differ in their approach. For instance, the Offender Management Model (OMM) of the National Offender Management Services (NOMS) in England and Wales favors an end-to-end approach and stresses well-planned case management and continuity of care (Maguire & Raynor, 2017). Integrated Offender Management (IOM) emphasizes multi-agency collaboration between prison services, the police, probation, local authorities, healthcare institutions, housing services, voluntary and community organizations, and other agencies, who should cooperate within the locality of the offender (Hadfield et al., 2020). The desistance paradigm of OM highlights the individual needs of offenders and the personal prisoner-professional relationships (McNeill, 2006).

Despite these different approaches, there is general consensus within the OM framework that professional assistance is crucial in managing or supporting the successful resettlement of prisoners. Multiple studies confirmed that case management and individual-level assistance (Day et al., 2012; Kendall et al., 2018; Visher et al., 2017) as well as in-prison support by community-based professionals, such as by parole officers (Bares & Mowen, 2020), can be useful in pre-release planning and post-release outcomes. Moreover, the systematic review of Hadfield et al. (2020) found that involvement of voluntary and community organizations or mental health services could be beneficial for addressing prisoners’ diverse needs.

The next question that arises concerns the *type of needs* that this team of professionals should address. In recent years, there is growing attention for the protective factors and destabilizers, or what we call *reintegration needs*, such as employment, housing, finances, healthcare, and valid identity documents. This increasing focus on reintegration needs was, in part, a reaction to the risk paradigm. It was argued that reintegration needs such as housing, economic stability, and healthcare should also be considered to reduce recidivism (Taxman & Caudy, 2015; Taxman & Smith, 2020) in addition to typical risk factors, such as antisocial attitudes (Andrews & Bonta, 2006). Moreover, within the desistance paradigm, tackling reintegration needs is seen as an important precondition for prisoners to work on a positive non-criminal identity and social position (Maguire & Raynor, 2006; McNeill, 2006; Ward et al., 2007). Furthermore, overly focusing on the individual deficits and potential risks of prisoners disregards what individuals say they need to desist from crime (Maguire & Raynor, 2006). Yet, previous research found that reintegration needs were often ignored in prisons, even though they could contribute to lowering recidivism and were of great concern to incarcerated and released persons (Bonta & Wormith, 2013; Petersilia, 2000), irrespective of their “risk level” (Scheirs, 2016).

In sum, according to the OM framework, it is important that the reintegration needs of all prisoners are assisted by a network of prison-based and community-based professionals, in order to remove barriers that often hamper the process of reintegration. In addressing these reintegration needs, individual-level support and continuity of care are considered vital.

## Offender Management in Dutch Prisons

The abovementioned OM principles and the growing attention to reintegration needs are also visible within the Dutch rehabilitation policy. According to the rehabilitation principle of the Dutch Penitentiary Principles Act (Pbw), a custodial sentence not only serves a retributive purpose, but should also aim at reintegration and pre-release planning. For the past two decades, the Dutch Custodial Institutions Agency (DJI) has emphasized five reintegration needs that are considered crucial for successful reintegration and post-release outcomes: employment, finances, housing, healthcare, and valid identification documents (DJI, 2019; Van Duijvenbooden, 2016).

To assess and monitor these reintegration needs, every prisoner is assigned a prison-based case manager and a mentor. The case manager is responsible for intake assessments with all prisoners *within the first 2* *weeks* of imprisonment. Moreover, the case manager functions as coordinator of the reintegration process and should transfer case information in the pre-release phase to community-based professionals (DJI, 2019). The mentor is a correctional officer who is expected to talk to prisoners at least *every other week* (Inspectorate of Justice and Security, 2018). Additionally, DJI made formal agreements with community-based professionals in how to provide throughcare and aftercare to prisoners (DJI, 2019). Current policy states that prison-based professionals, together with a parole officer and a municipal officer, need to discuss a reintegration plan with a prisoner *within 4* *weeks* of entry into prison (DJI, 2019). Parole officers usually assist prisoners at the start and at the end of imprisonment, carrying out supervisory tasks (e.g., court related advice), while also paying attention to reintegration needs (e.g., job referrals; Geenen et al., 2020). Municipal officers, who work at the municipality of origin or return, are responsible for providing access to community resources (e.g., valid ID) and for preparing prisoners for their return into the community (e.g., housing; DJI, 2019). Finally, community-based health- and care professionals are expected to make discharge plans for upon release concerning psychological and physical wellbeing, and volunteers of voluntary organizations often provide social services (e.g., job training, financial housekeeping, and housing options; Buysse et al., 2018; Kuis et al., 2015).

## Previous Findings on Professional Assistance in Prisons

Up until this point we described what professional reintegration support in prisons ideally looks like according to the OM framework. Based on previous literature, however, we know that in practice, professional assistance not always matches OM policies. For instance, the HM Inspectorates of Probation and Prisons in England and Wales, who evaluated the OMM of the NOMS, found that there is infrequent personal contact between the offender manager and prisoners, that there is limited in-prison involvement by community-based professionals, and that only high-risk offenders tend to receive assistance (Maguire & Raynor, 2017). Moreover, prison-based professionals often seem to lack the knowledge to implement offender management strategies, while community-based professionals, who are usually more trained in offender management, are more distant and often experience barriers to full inclusion (Maguire & Raynor, 2017). In general, there is often disappointment in what OM programs have achieved (Bullock & Bunce, 2020; Hollin et al., 2004; Maguire & Raynor, 2017).

Studies among professionals confirm this image. These often conclude that limited resources or limited time prevents them from seeking contact with prisoners (e.g., Hanrath et al., 2019; Petersilia, 2000; Plaisier et al., 2016; Turley et al., 2011). Moreover, professionals do not always seem to adjust their level of assistance to the reintegration needs of prisoners. For example, although the study of Viglione et al. (2015) was about *post-release* supervision, they found that probation officers often talked with prisoners about reintegration needs such as employment, housing, finances, and physical health, but that their supervision strategies were not adjusted to those assessed needs.

The lack of professional assistance has also been underscored by prisoners themselves. Interview studies in England and Wales found that prisoners reported that they remained invisible, were unable to reach their parole officer, and did not receive appropriate professional support (Bullock & Bunce, 2020; Crewe & Ievins, 2021). Large-scale research in the US found that about half of the surveyed serious and violent offenders reported pre-release contact with a case manager (Hamilton & Belenko, 2016; Visher et al., 2017), which was even lower among individuals who were not selected for participation in a reentry program funded by the Serious and Violent Offenders Reentry Initiative (SVORI; Lattimore & Visher, 2013). In the Netherlands, prisoners do not seem to discuss their reintegration plans very often with case managers or mentors (Plaisier et al., 2016) and they sometimes find it hard to reach their case manager (Hanrath et al., 2019). Yet, it is not known to what extent prisoners with specific reintegration needs receive relevant professional assistance. Finally, complaints about through- and aftercare and the absence of discharge and healthcare plans are well-documented (e.g., Abbott et al., 2016; Beerthuizen et al., 2015; Hopkin et al., 2018; McCauley & Samples, 2017).

Although previous literature revealed problems in resources, professional support, and through- and aftercare, these studies mostly described general shortcomings in support and did not link specific individual needs to assistance by relevant professionals. Furthermore, previous research typically focused on specific groups of prisoners such as violent offenders, contact with a single type of professional such as case managers or parole officers, or a single need such as drug problems or homelessness. Thus far, little is known about *in-prison* contact between prisoners with reintegration needs and *multiple* prison- and community-based professionals. To fill this gap, we set up the Dutch Prison Visitation Study (DPVS), part of the Life in Custody (LIC) study, to collect survey data among 4,309 prisoners and their (professional) visitors, across all 28 Dutch prisons. The current study contributes to the field of rehabilitation by focusing on an array of self-identified reintegration needs and offers a comprehensive assessment of the match between those needs and the level of assistance provided by relevant prison- and community-based professionals.

## Methods

### Data and Population

To examine the level of professional assistance provided to prisoners with reintegration needs, data from the Dutch Prison Visitation Study (DPVS), part of the Life in Custody Study (LIC-study), is used. The LIC-study is a largescale research project on the quality of life in all Dutch prisons and started in 2017 (Van Ginneken et al., 2018). The quality of prison life was measured by the Prison Climate Questionnaire (PCQ) and includes questions on the six domains of prison climate (relationships in prison, safety and order, contacts with the outside world, facilities, meaningful activities, and autonomy; for further details, see Van Ginneken et al., 2018).

The current study uses survey data from the second wave, held in February to May 2019, which included multiple DPVS questionnaires on the visitation experiences of both prisoners and their visitors, in addition to the standard PCQ. The DPVS-2019 did not only include regular visitors such as family and friends, but also professional visitors such as parole officers, municipal officers, health- and care professionals, and volunteers. With a team of forty research assistants, all prisoners were approached within 1 week per prison institution. Except for the psychiatric units, data was collected in all regimes, including pre-trial units.^1^ In addition, we approached all prison visitors at the entrance for 1 to 3 weeks per prison institution. However, for the present purpose, we focus on the prisoner point of view and use data on their self-reported reintegration needs and contact with professionals. Finally, administrative data was obtained, which contains background characteristics such as phase of imprisonment, regime, time served, and prisoner demographics.

At the time of data collection, 7,594 prisoners were held in custody, of whom 5,757 were able to participate. Prisoners were not able to participate when we were unable to approach them (e.g., they were released or transferred during the week of data collection or were placed in isolation). Other reasons not being able to participate included language barriers or psychiatric problems. In total, 4,350 unique questionnaires were collected among the target population, which was 76% of all handed out questionnaires in Dutch, English, Spanish, Polish, Turkish, and Arabic; 4,113 prisoners gave informed consent to link the survey to administrative data (95%). A further 196 surveys of newly arrived prisoners, initially not on our target list, were included in our research sample, which resulted in a total number of 4,309 unique participants. The respondents are representative of the total prison population with regard to time served, but participants turned out to be slightly older compared to the total prison population, women participated more often than men, and despite offering the survey in multiple languages, Dutch prisoners were overrepresented compared to non-Dutch prisoners.^2^

### Measures

#### Professional assistance

The dependent variables are dichotomous variables^3^ that reflect whether prisoners had face-to-face contact with a case manager or a mentor (the prison-based professionals), and with a parole officer, a municipal officer, a (health)care professional, or a volunteer (the community-based professionals) during the past 6 months of imprisonment or up to the point of data collection for prisoners who had served less than 6 months. Health- and care professionals included psychologists, psychiatrists, mental health professionals, drug treatment institutions, and other social care workers (other than volunteers). Volunteers included faith-based institutions and other social workers (voluntarily). For respondents who answered to all contact questions, an additional count variable was made for the overall level of assistance provided. Based on the distribution of this count-variable we categorized prisoners into a range from no assistance (0 professionals), a low overall level of assistance (1–2 professionals), a moderate overall level of assistance (3 professionals) to a high overall level of assistance (4–6 professionals).

#### Reintegration needs

Five dichotomous items were included to measure whether prisoners had (1) a job, (2) their finances in order, (3) a stable place to live, (4) a good health status,^4^ and (5) a valid identity document (ID) before they were imprisoned. A score of 1 on each of these variables means that prisoners had these needs (i.e., responded negatively on these items) prior to imprisonment. For respondents who answered to all needs questions, an additional count variable was made for the overall level of needs reported. Again, based on the distribution of the count-variable, prisoners were categorized into a range from no needs (0 needs), a low overall level of needs (1 need), a moderate overall level of needs (2–3 needs) to a high overall level of needs (4–5 needs).

#### Phase of imprisonment

Before we categorized prisoners into the *start, middle*, or *pre-release* phase, two groups were held separate from those three categories: (1) prisoners who served a maximum of 2 weeks at the point of data collection, because in accordance with the policy agreements it may take up to 2 weeks to do an intake assessment; and (2) prisoners with a total sentence length shorter than 4 months, because we were unable to distinguish meaningful phases of imprisonment.^5^ Following, the three categories were created based on time served and time to release. First, prisoners were considered in the *starting phase* when they served 2 to 6 weeks at the time of data collection. Given the Dutch policy, this group should have had contact with prison-based professionals (within 2 weeks) and additionally with a parole officer and municipal officer (within 4 weeks). The second group was in the *middle phase* and served longer than 6 weeks, but was not yet within 3 months of the release date. The third group was considered in the *pre-release phase* when they were within 3 months of the release date (Taxman et al., 2002).

### Analyses

Bivariate analyses were conducted to establish the match between reintegration needs and professional assistance, for the overall sample and split by phase of imprisonment. Given the bivariate nature of the analyses, information was deleted pairwise from each crosstabulation between a specific need and contact with a type of professional, to minimize the loss of information. We should keep in mind that group size and composition slightly differ for each crosstabulation.

## Results

Table 1 displays the level of reintegration needs and professional assistance of all participants. Altogether, 35% reported no needs, which means that 65% had at least one type of need. Most prisoners reported small (31%) or moderate (28%) overall levels of needs. Most commonly, prisoners reported having no employment prior to imprisonment (42%), followed by financial problems (27%), housing problems (23%), poor health (22%), and no valid ID document (11%). More than half of the participants reported contact with prison-based professionals, while contact with community-based professionals was substantially lower. Finally, most prisoners reported low (43%) or moderate (22%) overall levels of assistance.

**Table 1.** Descriptive Statistics (Total *N* ****=** **4,309).

	% Yes	Valid *N*
In-prison assistance		
* *Case manager	61	3,877
* *Mentor	59	3,937
* *Parole officer	42	3,979
* *Municipal official	17	3,971
* *(Health)care professional	25	3,976
* *Volunteer	17	3,964
Overall level of assistance		
* *Zero professional assistance	16	3,726
* *Low overall level of assistance	43	3,726
* *Moderate overall level of assistance	22	3,726
* *High overall level of assistance	19	3,726
Reintegration needs		
* *Unemployed	42	4,128
* *Financial problems	27	4,097
No (stable) place to live	23	4,143
* *Health problems	22	4,164
* *No valid ID	11	4,155
Overall level of reintegration needs		
* *Zero needs	35	3,981
* *Low overall level of needs	31	3,981
* *Moderate overall level of needs	28	3,981
* *High overall level of needs	6	3,981
Phase of imprisonment		
* *Total sentence length <4* *months	18	4,043
* *Time served <2* *weeks	4	4,043
* *Start: 2–6* *weeks	6	4,043
* *Middle: 6* *weeks–3* *months pre-release	56	4,043
* *Pre-release: <3* *months pre-release	16	4,043

### Reintegration Needs and no Contact With Any of the Professionals

**Figure 1.**
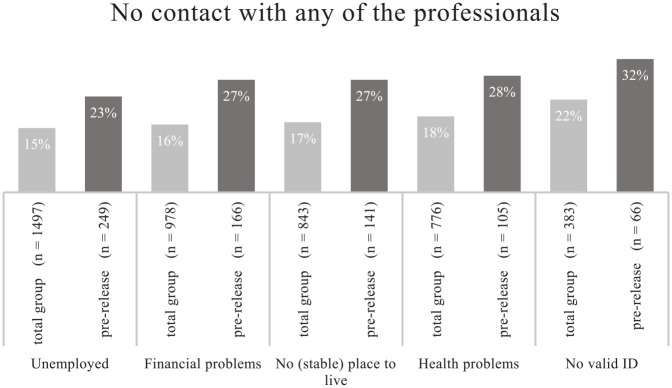
Percentage of prisoners with specific reintegration needs who had no contact at all, in total, and in the pre-release phase.

Next, we turn to the level of assistance reported by prisoners with reintegration needs. First of all, we examined how many prisoners with reintegration needs remained *invisible*. Figure 1 shows that 15% to 22% of the prisoners with employment, financial, housing, health, or ID needs reported no contact with any of the six professionals in the past 6 months of imprisonment or up until the point of data collection. For prisoners with health or ID needs, this turned out to be higher than for prisoners without such needs. This indicates that prisoners with health and ID needs were overlooked more often than prisoners without those needs. Moreover, additional analyses showed that there was overlap in having an ID need and reporting complex needs, meaning that prisoners with ID needs were more likely to report other needs as well, making it even more important that prisoners with ID needs are not overlooked.^6^

Additionally, Figure 1 specifies the results for prisoners who are in the pre-release phase, since it would be even more problematic if needs remain unassisted upon release. In the pre-release phase, 23% to 32% of the prisoners with needs reported no contact in the past 6 months. The number of prisoners that stayed under the radar in the pre-release phase is substantially higher than in other phases. However, there were no differences between prisoners with or without needs, meaning that assistance in the pre-release phase was generally low for both prisoners with and without needs. Yet, prisoners in the pre-release phase who entered prison with ID needs reported no contact with any professional relatively often, compared to prisoners with other needs.

Although the group sizes of prisoners who were in their pre-release phase and had a particular need were small at the point of data collection, around 30,000 Dutch prisoners are released each year. Thus, should this 23% to 32% hold on a larger scale, this would result in a substantive group of prisoners with needs that remains unassisted toward the end of imprisonment.

### Assistance by Relevant Professionals

Second, we examined whether prisoners with specific needs were assisted by *relevant professionals* (see Table 2). Given the pairwise deletion, Appendix A specifies the Valid *N* per crosstabulation. In absolute terms, prisoners with reintegration needs reported contact with prison-based professionals *more often* than with community-based professionals. For instance, 57% and 56% of the prisoners with a health need reported contact with a case manager or mentor respectively, whereas only 28% of the prisoners with a health need reported contact with a (health)care professional. Also, 59% and 53% of the prisoners with housing needs reported contact with a case manager and a mentor, whereas only 20% reported contact with a municipal officer. In other words, prisoners with needs have more contact with case managers or mentors than with the relevant community-based professionals.

**Table 2.** In-Prison Assistance per Reintegration Need.

	Prison-based				Community-based							
	Case Manager		Mentor		Parole Officer		Municipal Officer		(Health)care Professional		Volunteer	
	Contact (%)	OR	Contact (%)	OR	Contact (%)	OR	Contact (%)	OR	Contact (%)	OR	Contact (%)	OR
Unemployed												
No	61	1.02	59	.91	41	1.09	16	1.18	23	1.31**	16	1.15
Yes	62		57		44		18		28		19	
Financial problems												
No	62	.88	60	.82**	42	1.03	16	1.20	22	1.65**	16	1.42**
Yes	59		55		43		19		32		21	
No (stable) place to live												
No	62	.88	60	.74**	42	.99	16	1.31**	23	1.47**	17	1.25*
Yes	59		53		42		20		30		20	
Health problems												
No	63	.79**	60	.87	43	.88	17	.96	24	1.21*	18	.91
Yes	57		56		40		16		28		16	
No valid ID												
No	62	.77*	60	.59**	43	.75**	17	1.27	25	1.08	18	.83
Yes	56		47		36		20		26		15	
Prison-based professionals (any)					Community-based professionals (any)							
			Contact (%)	OR			Contact (%)		OR			
Needs (any)												
No			76	.85*			56		1.24**			
Yes			73				61					

** *p* ****<** **.01. *p ****<** **.05.

However, in relative terms, the odds ratios (OR) in Table 2 show an overall pattern of *less* contact with prison-based professionals and *more* contact with community-based professionals for prisoners with a specific need, compared to those without this need. For instance, prisoners who had no stable place to live were more likely to be assisted by (health)care professionals (OR = 1.47, *p* < .01), municipal officers (OR = 1.31, *p* < .01), and volunteers (OR = 1.25, *p* < .05), but less likely by mentors (OR = 0.74, *p* < .01), than prisoners who had a stable place to live. Likewise, prisoners with health problems were more likely to report contact with (health)care professionals, but less likely with case managers, than prisoners without health problems. Thus, in general, community-based professionals seek contact with prisoners less often than prison-based professionals, but when they do, they seem better focused on prisoners with relevant needs. Parole officers were an exception: they had contact with prisoners relatively often, but they were no more likely to be in contact with prisoners who had needs than those without needs, and were even *less likely* to be in contact with prisoners who had ID needs.

### Overall Level of Needs and Overall Level of Assistance

Third, we looked at the relationship between the *overall level of needs* and the *overall level of assistance* provided (see Table 3). Contrary to expectations, prisoners with the *highest* overall level of needs were significantly more likely to report no professional assistance at all, compared to prisoners with moderate and no needs (23% compared to 15%). More in line with expectations, prisoners with *moderate* needs more often reported a high overall level of assistance than prisoners with no needs (22% compared to 16%). There were no significant differences between other groups in relation to the overall level of needs and assistance.

**Table 3.** Overall Level of Reintegration Needs and Overall Level of Professional Assistance.

	No assistance (%)	Low assistance (%)	Moderate assistance (%)	High assistance (%)
Zero needs^a^	15^d^	46	23	16^c^
Low needs^b^	16	42	21	20
Moderate needs^c^	15^d^	41	22	22^a^
High needs^d^	23^a^,^c^	41	18	18

^a,b,c,d^*Group comparisons: p* < .05 with Bonferroni correction.

### Assistance Across the Phases of Imprisonment

**Figure 2.**
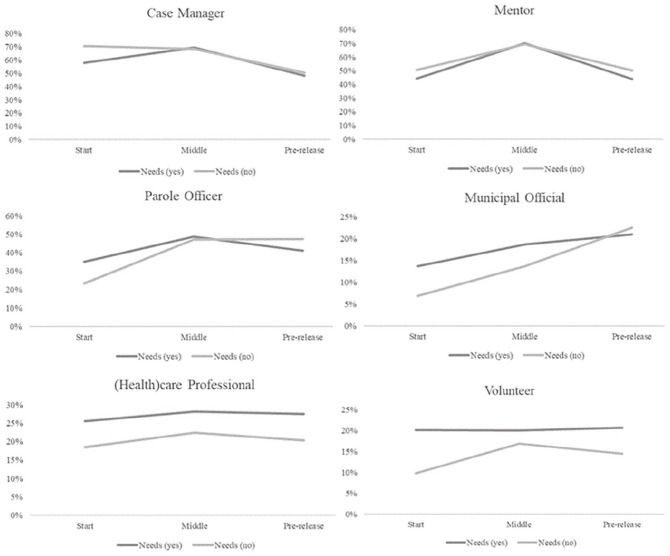
Percentage of prisoners with and without needs reporting contact with prison-based and community-based professionals per phase of imprisonment.

Fourth, we looked at the proportion of prisoners with needs who reported contact with each of the professionals at different *phases of imprisonment*. Appendix B presents the results for the two groups that were held separate (total sentence length <4 months and time served <2 weeks). Figure 2 presents the results for the start, middle, and pre-release phase and shows that contact with *prison-based professionals* is highest in the middle phase of imprisonment. These contact differences across phases were significant^7^ and contradict the expectation that prisoners would report more contact with prison-based professionals at the start due to the intake assessments. Yet, the higher amount of contact with mentors in the middle phase is in line with their expected monitoring tasks throughout detention.

Furthermore, looking at the differences between prisoners with and without needs, Figure 2 shows that the two lines run mostly parallel; any observed differences were not significant. In other words, there are no substantial differences between prisoners with or without needs in reporting contact with prison-based professionals across the phases.

Contact with *community-based professionals* also significantly differed across the phases of imprisonment, except for contact with (health)care professionals.^8^ Compared to the prison-based professionals, contact with community-based professionals was higher in the pre-release phase, or decreased less steeply. This is in line with the assumption that community-based professionals would help relatively often in the pre-release phase, when prisoners are preparing for their return to the community.

Comparing the two lines for prisoners with and without needs, prisoners with needs more often reported contact with parole officers and municipal officers at the start and middle phases of imprisonment, but this advantage was not sustained in the pre-release phase.^9^ Finally, prisoners with needs reported more contact with (health)care professionals and volunteers than prisoners without needs, and this contact was steady across the phases.

## Discussion

The importance of a personal approach to addressing reintegration needs is increasingly recognized. This has led to ambitious policy initiatives in different countries, including a Dutch version of integrated offender management that involves the collaboration of prison-based and community-based professionals. This study examined whether these professionals succeed in offering support to prisoners with reintegration needs. A few important findings emerged.

First of all, while most prisoners with needs are assisted by at least one professional, about one in five prisoners with needs remain invisible altogether. This means that intake assessments, individual reintegration plans, and follow-up mentoring still not fully succeed in preventing prisoners with complex, health, or ID needs from going unnoticed. Possibly, these prisoners have less human capital and face comparative disadvantage (Becker, 1962; Merton, 1968). Prisoners with complex needs might not be able to communicate their needs and to navigate through the complex professional networks of prison institutions (McSweeney & Hough, 2006). According to Hanrath et al. (2019), case managers were inclined to let prisoners take initiative after the first phase of imprisonment. However, not every prisoner might feel able to initiate contact, creating a gap in assistance between prisoners with and without complex needs. This refers to the idea of “the rich get richer and the poor get poorer” (Merton, 1968). In the Netherlands, for instance, a program implemented in 2012 got criticized for being available only to motivated prisoners without drug-related problems. Such criteria ignore the capacities that are needed to show motivation in the first place, such as help-seeking behavior and impulse control (Plaisier et al., 2016). Although since 2015 there is growing attention for needs complexity among prisoners (MoJ&S, 2017), the current study showed that it may still be helpful to offer prison-based professionals training on how to support prisoners with complex needs, especially those who may be least likely to take initiative in asking for help.

Fortunately, the majority of the prisoners did not remain invisible. More than half of the prisoners had contact with prison-based professionals, which seems somewhat higher than in previous international findings (Hamilton & Belenko, 2016; Visher et al., 2017); on top of that, community-based professionals were rather successful in visiting prisoners with relevant needs. This suggests that there is individual attention for prisoner needs and interagency collaboration between prison-based and community-based professionals to some degree. Information sharing could be one of the explanations that prison-based professionals are less often in contact with prisoners who have needs, handing those cases over to specialized help from community-based professionals. Given the potential value of specialized help from community-based professionals in preparing prisoners for release, it is recommended that in-prison assistance by community-based professionals is further promoted and funded. One Dutch initiative to do so were pilot tests in 2016 that placed parole officers within the prison walls of several institutions, making them operate in closer proximity to prisoners (Geenen et al., 2020). According to the parole officers, operating within the prison walls not only increased the amount of contact, but also made them more capable to focus on prisoner needs (RN, 2017).

This study showed that, similar to the prison-based professionals, parole officers were in contact with prisoners relatively often, but not necessarily with prisoners who had reintegration needs. Research has suggested that prison staff favor interacting with prisoners with less complex profiles (Bosma et al., 2018). Possibly, professionals who are in contact with prisoners most often, might in particular develop such preferences. Another possibility is that this is due to the dual task of prison-based professionals and parole officers, who are often in contact for multiple other reasons than reintegration assistance, such as maintaining a safe environment or court-related matters (Geenen et al., 2020). Yet, research has stressed the importance of social support by prison staff and parole officers in the reintegration of prisoners, rather than only focusing on risk management (Bares & Mowen, 2020; Doekhie et al., 2018; Maguire & Raynor, 2017; Ward et al., 2007).

Moreover, given the Dutch policy goals to contact every prisoner within 2 or 4 weeks for intake assessments and reintegration plans, and to prepare prisoners for release, we would have expected more contact at the start and end of imprisonment. Yet, previous research indicated comparable problems in intake assessments (Hamilton & Belenko, 2016; Lattimore & Visher, 2013; Schram et al., 2006) and pre-release planning (e.g., McCauley & Samples, 2017). One reason for the decreased amount of contact at the start of imprisonment, might be the overrepresentation in this phase of prisoners in pre-trial units. Although policy states that every prisoner should have had contact within 2 weeks, more attention possibly goes to prisoners who have a release date, which makes it easier to set up concrete reintegration plans.

Finally, our study found that prisoners with health needs were overlooked relatively often, also toward the end of imprisonment. Although (health)care professionals seemed rather attentive to prisoners with health needs, their limited levels of in-prison involvement in general still meant that less than one third of the prisoners with health needs reported contact with a community-based (health)care professional. These findings are in line with previous findings that discharge plans are often absent and that prisoners with health needs in particular are not always well-prepared in terms of continuation of healthcare upon release (e.g., Hopkin et al., 2018). High caseloads and lack of community resources are well-known obstacles to frequent contact and adequate pre-release support (Hanrath et al., 2019; Maguire & Raynor, 2017; Turley et al., 2011). Thus, good end-to-end management likely requires investment into staff and resources. A limitation of our study is that we did not distinguish between type of health needs, which limits conclusions about the specific assistance that would benefit prisoners.

A few other limitations are worth mentioning. First, we relied on self-report data. Therefore, the findings may not accurately reflect actual contact. For instance, participants may not have been familiar with the terms used in the survey to identify the professionals, or they may not have remembered contact moments with professionals. Including data from professionals on contact moments can offer a more complete picture. A further limitation of the study is that prisoners were asked to identify their needs *prior* to imprisonment. These reintegration needs, however, may change over time and imprisonment itself may create particular needs. This makes it important to maintain regular contact with prisoners during their sentence, regardless of pre-prison needs. On the other hand, particular needs might have been solved during imprisonment, between prison-entry, and the point of data collection. When using a measure of post-release expectations of reintegration needs, however, similar findings emerged. This suggests that a lack of contact with professionals could not be explained by the fact that pre-prison needs had been solved prior to the survey. Yet, future research should consider the timing of needs, changes in needs, and assistance throughout the phases of imprisonment. Another limitation is that the nature of the study, a survey, required the predetermination of categories of professionals and needs. While these categories were most prominent based on policy, it is likely that some professional assistance and needs were not included. Moreover, the binary answering options on either having a need or not do not allow for nuances. For example, previous research has suggested that job quality and stability matter in the protective role of employment (e.g., Ramakers, 2014). This means that prisoners who were employed prior to imprisonment, and therefore did not report an employment need, might still need guidance toward higher quality or more stable jobs. Thus, although the questionnaire was able to show the amount of prisoners reporting a need and receiving no assistance from a (relevant) professional, the comparisons to prisoners without needs should be interpreted more cautiously. Finally, although we used pairwise deletion to minimize information loss, prisoners with certain background characteristics might have been underrepresented in the crosstabulations. For instance, additional analyses showed that overall, prisoners with short sentences tended to have missing information on contact with professionals more often.

For future research it would be interesting to zoom in on the nature of contact and explanations for differences in contact. For example, the frequency and timing of contact may be related to readiness to change, human capital, age, gender, ethnicity, criminal histories, or contextual barriers experienced by community-based professionals. It would also be worthwhile to examine the degree to which contact with professionals is successful in addressing needs, and how satisfied prisoners are about this contact. This would also give further insight into the importance of reintegration needs in preventing future offending. In the present study we argued that targeting reintegration needs can contribute to lower future reoffending, on top of the typical risk factors such as criminal history, antisocial personalities, antisocial attitudes, and antisocial associates (Andrews & Bonta, 2006). Also, according to the desistance paradigm, resolving reintegration needs might enable the process of desistance (e.g., McNeill, 2006). Moreover, we made the case that prisoners with reintegration needs should be supported irrespective of their risk level. Yet, we do not wish to ignore the importance of addressing the typical risk factors in preventing future reoffending. Previous research has repeatedly showed that these typical risk factors (or criminogenic needs or “Big Four”) are related to the risks of recidivism (Andrews & Bonta, 2006). Thus, although we chose to avoid a risk-perspective on prisoner support, and use an offender-centered perspective instead, it would still be valuable for future research to include the criminogenic needs in terms of lowering the risks of recidivism. It is already known that prisoners with criminogenic needs are not always properly referred to in-prison programs (e.g., Long et al., 2019). Yet, it remains unclear to what degree prisoners with criminogenic needs are supported by a team of relevant professionals.

Overall, the current study has demonstrated the importance of mapping reintegration needs and assistance, in order to fulfil the promise of offender management. The promise of offender management, however, is less evident in countries beyond the western world. Whereas the NOMS in the UK and the Dutch rehabilitation policies include offender management strategies, in Latin America, for instance, rehabilitative policies are not common (Sanhueza et al., 2018). In most Latin American countries, the criminal justice system is punitive and focuses on retribution and control, without formal guidelines on prisoner support (Sanhueza et al., 2018; Villagra & Droppelmann, 2016). Reintegration efforts often depend on the goodwill of social workers and other professionals (Villagra & Droppelmann, 2016). Also, poor infrastructure and the fact that prison research is still developing in Latin America (Sanhueza et al., 2021) makes it difficult to get political and economic support for rehabilitative policies. Evaluating certain aspects of rehabilitative systems in Europe and in the USA, might help inform rehabilitative policies in countries that are beginning to develop these.

To that end, in the Netherlands, a few aspects of the rehabilitation system seem promising. For instance, the reasonable amount of contact with prison-based professionals combined with specialized reintegration support by community-based professionals, assumes that there is individual attention and interagency collaboration. Also, initiatives are undertaken regarding complex needs and in-prison involvement of community-based partners. Yet, in line with previous OM evaluations (Hollin et al., 2004; Maguire & Raynor, 2017), challenges still appear in end-to-end management, community-based involvement, and in reaching all prisoners with reintegration needs.

## Appendix

**Appendix A.** Number of Valid Cases per Crosstabulation in Table 2.

	Prison-based		Community-based			
	Case manager	Mentor	Parole officer	Municipal officer	(Health)care professional	Volunteer
Unemployed	3,807	3,868	3,909	3,897	3,902	3,889
Financial problems	3,781	3,836	3,880	3,873	3,877	3,864
No (stable) place to live	3,821	3,876	3,921	3,915	3,919	3,907
Health problems	3,815	3,873	3,917	3,909	3,914	3,900
No valid ID	3,825	3,881	3,928	3,920	3,925	3,912
	Prison-based professionals (any)			Community-based professionals (any)		
Needs (any)	3,873			3,924		

**Appendix B.** Contact With Professionals for Prisoners With and Without Needs for the Short Sentence and < 2 weeks Groups.

	Case manager		Mentor		Parole officer		Municipal officer		(Health)care professional		Volunteer	
	Contact (%)	OR	Contact (%)	OR	Contact (%)	OR	Contact (%)	OR	Contact (%)	OR	Contact (%)	OR
Total sentence length <4 months												
Needs (yes)	55	1.01	37	.71*	34	1.35	19	1.31	26	1.82**	14	1.36
Needs (no)	55		45		28		15		16		11	
Time served <2 weeks												
Needs (yes)	35	1.87	28	.98	36	1.24	21	1.33	26	.86	12	.67
Needs (no)	22		29		31		17		29		17	
	Valid *N*		Valid *N*		Valid *N*		Valid *N*		Valid *N*		Valid *N*	
Total sentence length <4** **months												
Needs (yes)	420		427		432		433		433		432	
Needs (no)	220		221		225		225		222		223	
Time served <2 weeks												
Needs (yes)	75		81		81		81		82		82	
Needs (no)	41		45		42		42		42		41	

** *p* ****<** **.01. **p* ****<.** **05.

## Notes

1. In the Netherlands, pre-trial detainees are incarcerated in separate units in the same prison institutions as convicted prisoners, and the same rules concerning professional support apply to them.

2. Age: [participants: *M* = 37.13, *SD* = 11.92 vs. the population: *M* = 36.76, *SD* = 11.82; *t*(4,349) = 2.063; *p* = .039]. Time served: [participants: *M* = 12.75, *SD* = 24.17 vs. the population: *M* = 12.39, *SD* = 23.35); *t*(4,349) = 0.407, *p* = .684]. Nationality: [χ^2^(1, *N* = 7,284) = 56.23, *p* < .000; effect size: OR = 1.56, 95% CI (1.39, 1.76)]. Gender: [χ^2^(1, *N* = 7,594) = 47.16, *p* < .000, effect size: OR = 2.19, 95% CI (1.74, 2.76)].

3. The items were originally measured by the frequency of contact, including answering options “never,” “monthly,” “weekly,” or “daily” for the prison-based professionals and 0 times, 1 to 2 times, 3 to 5 times, or more than 6 times for the community-based professionals. However, very few prisoners reported high frequencies of contact and ordinal analyses showed violation of the parallel line test [prison-based: χ^2^(24, *N* = 3,589) =  55.43, *p* < .01; community-based: χ^2^(24, *N* = 3,724) = 88.61, *p* < .01)], which means that the estimates are not consistent over the ranked responses on contact frequency. Therefore, and also because there seems to be a fair amount of prisoners who report no contact with the various types of professionals, we decided that it would be more meaningful and straightforward to interpret whether prisoners reported contact with either one of these professionals during the past 6 months or not.

4. This item was originally measured by a 1 to 5 Likert-scale. To keep interpretations consistent for the five reintegration needs, we dichotomized this variable. Prisoners who rated their health as 1 or 2 were considered in poor health, compared to 3, 4, and 5, who were considered in average or good health.

5. For instance, prisoners with a total sentence length of 3 months would be in their starting phase as well as in their pre-release phase.

6. The results of additional analyses are available from the corresponding author on request.

7. Case manager, phase differences: χ^2^(4, *N* = 3,604) = 149.67, *p* < .01; Mentor, phase differences: χ^2^(4, *N* = 3,650) = 292.83, *p* < .01.

8. Parole officer, phase differences: χ^2^(4, *N* = 3,694) = 73.55, *p* < .01; Municipal officer, phase differences: χ^2^(4, *N* = 3,686) = 16.06, *p* < .01; (Health)care professional, phase differences: χ^2^(4, *N* = 3,692) = 3.05, *p* > .05; Volunteer, phase differences: χ^2^(4, *N* = 3,679) = 13.07, *p* < .05.

9. Parole officer, start: OR = 1.79, 95% CI (1.00, 3.21), *p* = .052; Parole officer, pre-release: OR = 0.77, 95% CI (0.55, 1.08), *p* *>* .05; Municipal officer, middle: OR = 1.45, 95% CI (1.14, 1.83), *p* < .01; Municipal officer, pre-release: OR = 0.91, 95% CI (0.61, 1.37), *p* > .05.
